# Assembly-promoted repeatable enhancement of photoluminescence from cesium lead tribromide nanocubes under light illumination†

**DOI:** 10.1039/d4na00665h

**Published:** 2024-10-15

**Authors:** Shota Hashimoto, Hiroto Watanabe, Yoshiki Iso, Yuya Oaki, Tetsuhiko Isobe, Hiroaki Imai

**Affiliations:** a Department of Applied Chemistry, Faculty of Science and Technology, Keio University 3-14-1 Hiyoshi, Kohoku-ku Yokohama 223-8522 Japan hiroaki@applc.keio.ac.jp

## Abstract

A repeatable enhancement of the photoluminescence (PL) from CsPbBr_3_ nanocubes (NCs) is promoted by the assembling of NCs. The PL quantum yield (QY) of ordered NC arrays increases with photoirradiation and decreases in the dark. The repeatable enhancement of the PLQY cannot be observed from isolated NCs. The nanospaces between NCs in the ordered arrays allow a reversible change in thermally stimulated desorption and photo-induced adsorption of surface ligands that affect the PLQY.

## Abstract 2

A repeatable enhancement of the photoluminescence from CsPbBr_3_ nanocubes under photoirradiation is promoted by their ordered assembly.

## Graphical abstract

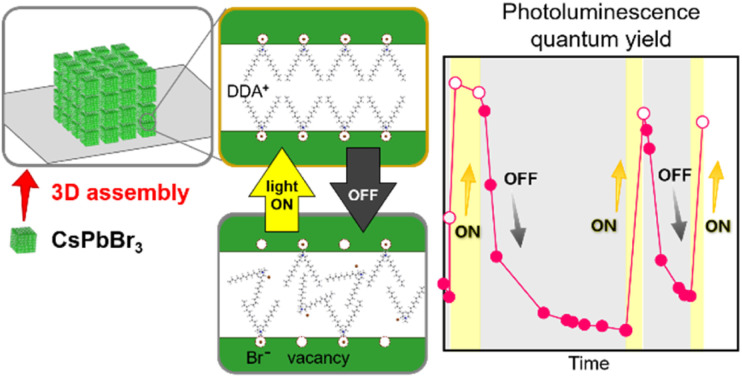

Perovskite cesium lead halide (CsPbX_3_: X = Cl, Br, I) nanocubes (NCs) are now becoming one of the most promising candidates of quantum dot (QD) fluorophores.^1–5^ High photoluminescence (PL) quantum yields (QY) and high color turnabilities in a wide visible spectral range are the advantages of these materials. In general, however, NC fluorophores show a serious decrement in the PLQY especially under continuous photoirradiation due to the aerobic oxidation and crystal growth of nanoscale grains.^6–11^ Several approaches, such as coverage with specific surface capping agents,^12–14^ impregnation into polymer matrices,^15,16^ encapsulation in molecular bottlebrush trilobes,^17^ and fabrication of silica–polymer composites,^5,18^ were developed to improve the stability of CsPbBr_3_ NCs. Recently, our research group demonstrated the photoactivation and improved photostability of CsPbBr_3_ NCs by constructing an ordered two-dimensional (2D) array with a specific surface-capping agent.^19^ A high PLQY (*ca.* 60%) of NCs in the 2D arrays was maintained for more than 4000 h under continuous photoirradiation. In this work, we found a repeatable enhancement of PL from CsPbBr_3_ NCs by their assembly on a substrate with the photo-induced adsorption of surface ligands (Fig. 1).

**Fig. 1:**
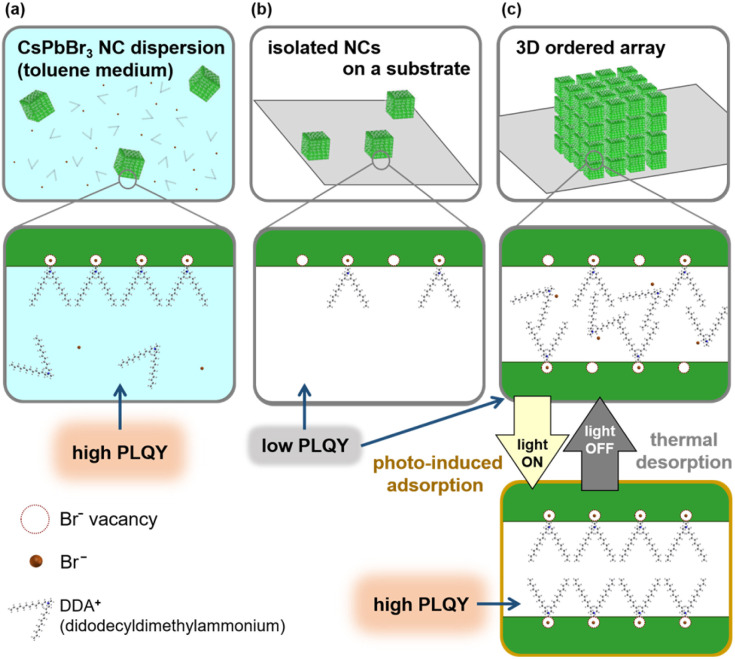
Schematic illustrations of the degradation and repeatable photoactivation of PL from CsPbBr_3_ NCs with the adsorption/desorption of surface ligands. (a) Dispersion of toluene, (b) isolated NCs on a substrate, and (c) 3D array on a substrate.

The photoactivation of QDs was reported by several researchers.^8,20,21^ The mechanisms for the photoactivation of QDs were categorized into four types,^20^ such as defect reduction mediated by heat induction,^22,23^ the adsorption of water molecules,^10,24,25^ surfactant rearrangement,^26,27^ and photo-oxidation.^28,29^ However, the photoactivation of CsPbBr_3_ NCs in the array was not ascribed to these mechanisms because the phenomenon was not observed in isolated NCs. This suggests that the assembly states have a key role for photoactivation and photostability.

The rectangular shapes of NCs realize the formation of highly ordered 2D and three-dimensional (3D) arrays. Ordered assemblies in which the crystal lattices are arranged in the same orientation are fabricated by rectangular nanocrystals, such as NCs.^30–34^ Such single-crystal-like structures consisting of iso-oriented nanometric building blocks are called mesocrystals.^35–37^ These unique structures have attracted much attention because of their specific functionalities, including mechanical,^38–41^ electrochemical,^30,42,43^ magnetic,^31,32,44^ dielectric,^33,45^ sensing,^34,46^ and photocatalytic^36,47^ properties. Rectangular nanoblocks of metals and metal compounds that are dispersed in a liquid medium are accumulated through self-assembly by various techniques using sedimentation, dispersibility changes, evaporation, and other phenomena.^35,48–50^ In our previous work, the controlled fabrication of 2D and 3D arrangements of NCs and nanocuboids was demonstrated using evaporation-driven self-assembly.^51,52^ The PL property was reported to be influenced by the 3D assembly of CsPbBr_3_NCs.^19,53^

In the present study, we focused on the photoactivation of CsPbBr_3_ NCs in the 2D and 3D arrays. As shown in Fig. 1, the PLQY decreases by the removal of dispersion liquid media containing ligand molecules. However, we found that the PL performance of CsPbBr_3_ NCs without dispersion liquid media was influenced by the assembly states. Although a low PLQY of isolated NCs on a substrate was not changed regardless of photoirradiation, an increase of the PLQY with photoirradiation and a subsequent decrease with room-temperature annealing were repeatably observed on NCs in the ordered arrays. According to the activation energy analysis and PL lifetime studies, the repeatable change in the PLQY was deduced to be based on the photo-induced adsorption and thermally stimulated desorption of the surface-capping agent. The reservation of ligand molecules in the nanospaces between adjacent NCs is essential for repeatable adsorption/desorption.

Here, CsPbBr_3_ NCs were prepared by the hot-injection method reported in the previous study^1^ (see the ESI† for the detailed experimental procedure). The surface-capping agents were exchanged from oleic acid (OA)/oleyl amine (OLA) to didodecyldimethylammonium bromide (DDAB). As shown in a TEM image (Fig. S2 in the ESI†), well-dispersed CsPbBr_3_ NCs were obtained at an average side length of 9.8 ± 1.1 nm. We deposited the CsPbBr_3_ NCs on a silica glass substrate in three different states, such as isolated grains (Fig. 2a), 2D (Fig. 2b), and 3D (Fig. 2c, d) ordered assembled arrays. From TEM images, the average inter-cube distance of the NC array was estimated to be about 2–3 nm. The NCs on the glass substrate were covered with another silica glass plate that was surrounded with ultraviolet (UV) curing resin to prevent contact with oxygen in the air.

**Fig. 2:**
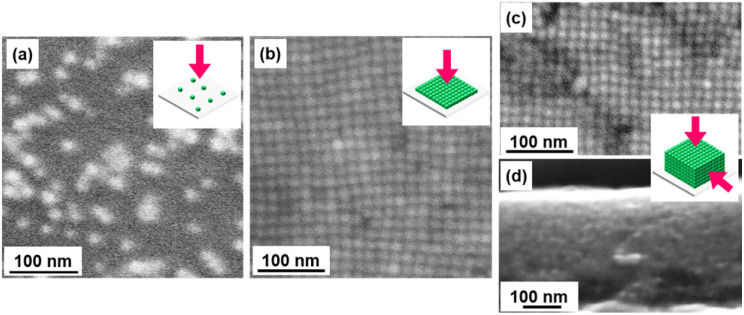
SEM images of (a) isolated NCs and (b) 2D and (c, d) 3D ordered assembled arrays with their schematic illustrations.

UV-visible absorption and PL spectra for CsPbBr_3_ NCs in a dispersion and in three different states on the substrate are shown in Fig. S3 in the ESI.† For a dispersion, the wavelength at the maximum PL intensity (*λ*_max_) was 515 nm (2.41 eV) with the PLQY 64.9% by excitation at 450 nm. The *λ*_max_ from randomly deposited isolated NCs was 513 nm (2.42 eV) with the PLQY 19.4 ± 0.1% (*n* = 2). The PL spectra from the CsPbBr_3_ NCs are agreed with those depending on their size.^54^ The values of *λ*_max_ from NCs in 2D and 3D assembled states show redshifts to 519 nm (PLQY: 18.1 ± 7.6%, *n* = 13) and 528 nm (PLQY: 16.0 ± 7.8%, *n* = 25), respectively. The spectral change is ascribed to the formation of mini-bands that originate in a quantum-resonance effect that occurs for the assembled states. The decrease in PLQY was observed after the removal of dispersion liquid media. Since the free DDAB in the dispersion easily adsorbs to the NC surface in adsorption equilibrium, the NC dispersion showed high PLQY owing to stable surface passivation (Fig. 1a). However, the DDAB thermally desorbed from the NC surface without dispersion liquid media, resulting in a decrease in the PLQY (Fig. 1b and c). In particular, free DDAB around isolated NCs was removed along with the dispersion liquid media by the spin-coating process.

Fig. 3 shows the PLQY change of the 3D array and the isolated NCs under continuous photoirradiation at 468 nm. The PLQY of the 3D array reaches its maximum within ∼3 h. In contrast, a trace increment in the initial PLQY was observed for the isolated NCs. Therefore, assembled states are essential for the present photoactivation. As described in our previous study^19^ and shown in Fig. S4 in the ESI,† we also observed an initial increment in the PL intensities under photoirradiation of the 2D assembled states.

**Fig. 3:**
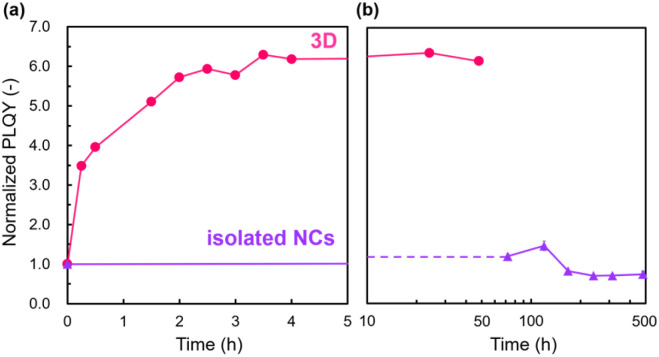
Time courses of the PLQY of isolated NCs and 3D ordered assembled arrays under continuous photoirradiation at 468 nm within (a) 5 and (b) 500 h.

Fig. 4 shows the PLQY changes of the 3D assembled array with and without photoirradiation. The PLQY increased up to 68.3 ± 2.2%, (*n* = 6) under photoirradiation and decreased in the dark state. These changes in the PLQY with the ON/OFF of photoirradiation were confirmed to be repeatable. Fig. S5 in the ESI† shows that the *λ*_max_ of PL was not changed after the PLQY changes. Thus, the states of NCs after photoirradiation were identical to those after room-temperature annealing. We also observed similar increases and decreases in the PLQY for the 2D arrays (Fig. S5 in the ESI†).

**Fig. 4:**
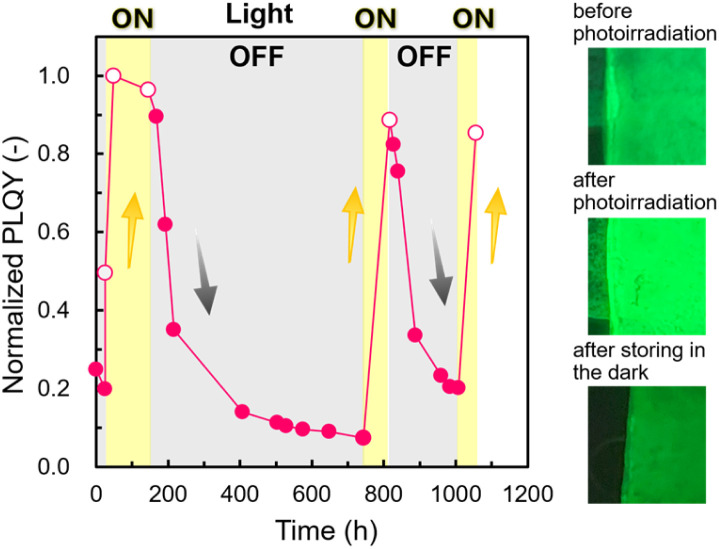
The PLQY change of the 3D ordered assembled arrays of CsPbBr_3_ NCs with and without photoirradiation. Open and filled circles indicate changes under irradiation and in the dark state, respectively.

Whereas the PLQY of the assembled states increased with photoirradiation for several hours (Fig. 3), the decay of the PLQY occurred gradually over 600 h in the dark (Fig. 4). Photoactivation owing to defect annihilation with the photo-induced ion migration was reported for organohalide lead perovskites.^55,56^ In this case, however, the increment and decrement of the PLQY were completed within ∼600 and ∼4000 s, respectively. The timescales shown in the previous study are one to three orders of magnitude faster than those for the PL in the present work. Therefore, the activation and decay mechanisms of the present PL changes cannot be ascribed to ion migration.

To clarify the origin of the PLQY variation, we measured the temperature dependence of the PL decrement in the dark. As shown in Fig. S6 in the ESI,† PL decrement rates increased by elevating temperature. According to the temperature dependence, which was fitted with a pseudo-second-order Arrhenius equation, the activation energy of the present PL decrement was determined to be 42.1 kJ mol^−1^, which corresponds to common physical adsorption/desorption reactions.^57^ Since the as-prepared CsPbBr_3_ NCs contain surface defects, the surface-capping agents, such as DDAB, are required to eliminate the trapping centers to achieve a high PLQY. Therefore, the PLQY decrement is ascribed to the thermally stimulated desorption of the surface-capping agent. Recently, the decrease in the PL intensity of CsPbBr_3_ was reported to occur due to the thermal desorption of surface ligands (OA/OLA).^17,18^ Since the timescale of the decrement of the PLQY in the present study is similar to that reported in the previous articles, the change in the PL intensity is ascribed to the thermally stimulated desorption of the ligands confined in the inter-cube spaces. Moreover, the decrement in the PL lifetime of CsPbBr_3_ was reported in the absence of the ligands. The PL lifetime change is originated from the formation of surface traps along with the desorption of ligands. In contrast, the increment in the PL lifetime of CsPbBr_3_ NCs was induced by the adsorption of DDAB through reduction of the surface traps.^16^ Therefore, we measured the PL lifetime of dilute dispersion of NCs and 3D assembled arrays stored in the dark and after photoirradiation to verify the mechanism of the reversible PLQY changes. As shown in Fig. S7 in the ESI,† the average PL lifetime of the 3D array decreased (10.5 ns) from that for the dilute dispersion (15.2 ns) in the dark and increased under photoirradiation (22.6 ns). We observed reversible increase and decrease in the PLQY with a change in the PL lifetime. DDAB is deduced to desorb thermally in the dark and re-adsorb under photoirradiation.

The PL enhancement under photoirradiation and decrement in the dark were not observed for isolated NCs. Therefore, the fabrication of an ordered assembly is the key factor for achieving repeatable reactions. In highly ordered states, DDAB molecules were encapsulated in the nanospaces between the NCs. However, the desorption of DDAB molecules (∼1.6 nm) would occur in the dark because of the large inter-cube distance (∼3 nm). Since the molecules are still reserved in the nanospaces, re-adsorption is achieved under photoirradiation (Fig. 1).

The adsorption of DDAB could be induced by the change in the surface charges due to electron accumulation on the surface traps under photoirradiation. As reported in the previous report,^58^ CsPbBr_3_ NCs produced by the hot-injection method are terminated with Br-rich surfaces, which contain Br^−^ vacancies. Photo-induced electrons under blue-light illumination are trapped at Br^−^ vacancies (Fig. 5). Therefore, the strong adsorption of cationic DDAB is expected on the negatively charged surface with the accumulation of photo-induced electrons.

**Fig. 5:**
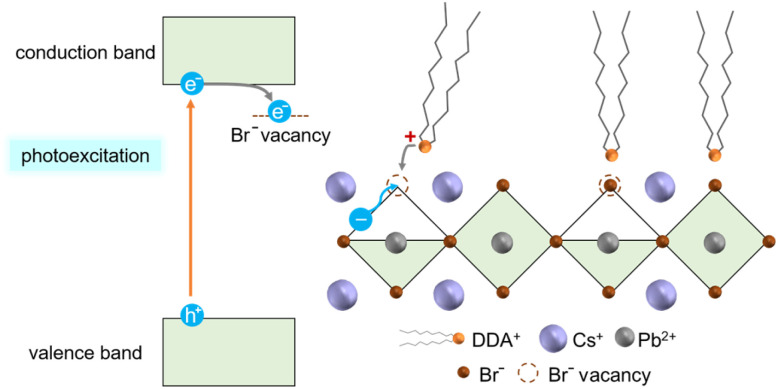
Schematic illustration of the photo-induced adsorption of DDAB on the surface of the NC ordered array under blue-light illumination.

Since DDAB molecules are tightly adsorbed on the surface of the NCs, undesired crystal growth and surface oxidation are prevented.^12,59^ Therefore, photo-induced ligand adsorption and molecular encapsulation in the nanospaces in the ordered arrays of NCs play key roles in the high photostability of CsPbBr_3_. The 3D ordered array shows high PLQY of about 60% under continuous photoirradiation over 6000 h (Fig. S8 in the ESI†).

In the present study, we fabricated highly ordered 2D and 3D assemblies of CsPbBr_3_ NCs and discovered a repeatable PL enhancement under photoirradiation. The kinetic analyses suggest that this phenomenon originates from photo-induced adsorption and thermally stimulated desorption of the surface-capping agent. The molecular encapsulation in the nanospaces in the NC assembly allows repeatable adsorption/desorption. The fabrication of the assembled structure is effective to design QD-based fluorophores with high photostability.

## Data availability

The authors declare that the data supporting the findings of this study are available within the paper and its ESI† file. Raw data files are available from the corresponding author upon reasonable request.

## Conflicts of interest

There are no conflicts to declare.

## Supplementary Material

NA-OLF-D4NA00665H-s001
