# Supplementary material for: Assembly-promoted repeatable enhancement of photoluminescence from cesium lead tribromide nanocubes under light illumination
Source: Nanoscale Adv. 2024 Oct 15;6(22):5563–7. doi: 10.1039/d4na00665h (PMC11480829; doi:10.1039/d4na00665h)
Supplement: NA-006-D4NA00665H-s001 [file NA-006-D4NA00665H-s001.pdf]

Supplementary Information (SI) for Nanoscale Advances.
This journal is © The Royal Society of Chemistry 2024

Supporting information

Assembly-promoted repeatable enhancement of photoluminescence from cesium lead tribromide nanocubes under light illumination

Shota Hashimoto,[a] Hiroto Watanabe,[a] Yoshiki Iso,[a] Yuya Oaki,[a] Tetsuhiko Isobe[a] and Hiroaki Imai[a*]

[a] Department of Applied Chemistry, Faculty of Science and Technology, Keio University, 3-14-1 Hiyoshi, Kohoku-ku, Yokohama 223-8522, Japan.

E-mail: hiroaki@applc.keio.ac.jp

Table of contents

Materials S2

Experimental procedures S2

Figure S1. Schematic illustrations of preparation methods for various arrays of CsPbBr3 NCs S3

Figure S2. TEM images and size distributions of $CsPbBr_3$ NCs S4

Figure S3. UV-visible absorption spectra and PL spectra of $CsPbBr_3$ NCs S4

Figure S4. Time courses of PLQY of isolated NCs and 2D ordered arrays S5

Figure S5. The PLQY changes and the PL spectra of the 3D and 2D ordered arrays S6

Figure S6. The PLQY decrement in the dark at different temperature of 3D ordered arrays S7

Figure S7. Time resolved PL decays and the fitted curves of $CsPbBr_3$ NCs S8

Figure S8. Time courses of PLQY of isolated NCs and 3D ordered assembled arrays S8

Materials

Cesium carbonate (Cs_2CO_3, 98.0%, Kanto Chemical), lead bromide ($PbBr_2$, 99.999%, Thermo Scientific), 1-octadecene (90.0%, Kanto Chemical), oleic acid (OA, 65.0+%, FUJIFILM Wako Pure Chemical), oleylamine (OLA, 70%, Aldrich), didodecyldimethylammonium bromide (DDAB, 98%, Sigma-Aldrich), toluene (99.5%, Kanto Chemical), *tert*-butyl alcohol (99.0%, FUJIFILM Wako Pure Chemical), ethyl acetate (98.0%, Tokyo chemical industry). All the materials were used as purchased.

Experimental procedures

Preparation of $CsPbBr_3$ NCs and ligand exchange

The perovskite NCs were prepared by using the hot-injection method reported in the previous study.[1] As starting materials, we used Cs_2CO_3 and $PbBr_2$ without further purification. A mixture of 1-octadecene (40.0 cm^3) and oleic acid (2.5 cm^3) containing Cs_2CO_3 (2.50 mmol) was vacuum-dried for 1 h at 120 °C and then heated to 150 °C under an inert gas atmosphere until the powder was completely dissolved. This Cs-oleate precursor solution was preheated at 100 °C before injection. A mixture of 1-octadecene (5.0 cm^3) and $PbBr_2$ (0.188 mmol) was vacuum-dried for 1 h at 120 °C and then a mixture of oleylamine (0.50 cm^3) and oleic acid (0.50 cm^3) was added to this solution under an inert gas atmosphere. After heating the mixture solution to 165 °C, we quickly injected the preheated Cs-oleate precursor solution into the mixture. After 5 seconds later, the resultant solution was then cooled in an ice-water bath. The aggregated NCs were separated by centrifugation at 13500 rpm for 5 minutes after adding *tert*-butyl alcohol (5.0 cm^3). The purified NCs were dispersed in toluene.

The ligand exchange was performed according to a previous article.[2] We added OA (0.10 cm^3) and 0.05 M DDAB (0.20 cm^3) to a $CsPbBr_3$ NCs dispersion (1.0 cm^3) with a concentration of 1.87 g dm^{-3}. After the mixture was stirred for 3 minutes at room temperature, the ligand-exchanged NCs dispersion was centrifuged with adding ethyl acetate (1.0 cm^3) at 13500 rpm for 5 minutes. The purified $CsPbBr_3$ NCs were redispersed in toluene.

Preparation of various $CsPbBr_3$ NC arrays

Following experimental preparation methods were shown in Figure S1. DDAB-covered $CsPbBr_3$ NCs were dispersed in organic medium (toluene : hexane = 1 : 1 (v/v)). Isolated NCs were deposited on a collodion film that was spin-coated on the surface of a silica glass substrate to prevent aggregation of NCs. 0.05 cm^3 collodion-ethanol solution (1:1 volume) was prepared and was deposited on a silica glass substrates (8 × 8 mm) by the spin-coating method (1000 rpm, 30 s). Subsequently, 0.01 cm^3 NCs dispersion was deposited on the collodion film by the spin-coating method (1000 rpm, 30 s).

Highly ordered 2D monolayer arrays of NCs were accumulated on a silica glass substrate by a convective self-assembly method with evaporation of the dispersion medium according to a previous work[3]. Oleic acid (0.01 cm^3) was added to the NCs dispersion (toluene : hexane = 1 : 1 (v/v)) to prepare 0.5 cm^3 dispersion in 6 cm^3 glass vials. Silica glass substrates (7 × 16 mm) were washed with acetone by ultrasonication for 30 min and then they were placed into the dispersion in the glass vials. The dispersion spread on the glass substrate after immersion and the NC assemblies were then deposited on the substrate through the liquid film recession by the evaporation of the dispersion medium.

Ordered 3D multilayer arrays of NCs were accumulated on a silica glass substrate by the slow evaporation rate of the dispersion medium. Silica glass circular substrates (15 mm in diameter) was washed with acetone by ultrasonication for 30 min and then they were laid into 6 cm^3 glass vials. 4 cm^3 NCs dispersion (toluene : hexane = 1 : 1 (v/v)) was added into the glass vials and the NC assemblies were deposited on the glass substrate through slow evaporation of the dispersion medium.

Blue LED irradiation of the NC arrays

Light irradiation was performed according to a previous work.[4] The NC arrays on the silica glass substrate were covered by a silica glass plate with ultraviolet (UV) curing resin (Bondic BD-SKEJ) to prevent degradation owing to water and oxygen in the atmosphere. The NC arrays were irradiated using a flat-panel blue LED (Nissin Electronics TE-4556). The light wavelength and irradiance of the blue LED were 468 nm and 48.5 W m^{-2}, respectively.

To examine the change on the PL property with intermittent photoirradiation, photoirradiation of the NC arrays and storage in the dark were performed repeatedly. In addition, to measure the temperature dependency of PL decrement, the NC arrays were stored in the dark at different temperatures (–10°C, 25°C, 40°C, and 60°C) after long enough time to increase its maxima with photoirradiation.

Characterization

The UV–visible absorption spectra of dispersion and film samples were measured using UV/visible/near-infrared optical absorption spectrometers (JASCO V-750 and V-570). PL spectra were measured using a fluorescence spectrometer (JASCO FP-8500). PLQYs of dispersion and film samples were measured using an absolute quantum yield spectrometer (Hamamatsu C9920-02G). The morphology and the crystalline phase of the NCs were monitored using transmission electron microscopy (TEM, FEI Tecnai G^2), scanning electron microscopy (SEM, JEOL JSM-7100). The PL lifetime was measured using a fluorescence lifetime spectrometer (Hamamatsu Quantaurus-Tau).

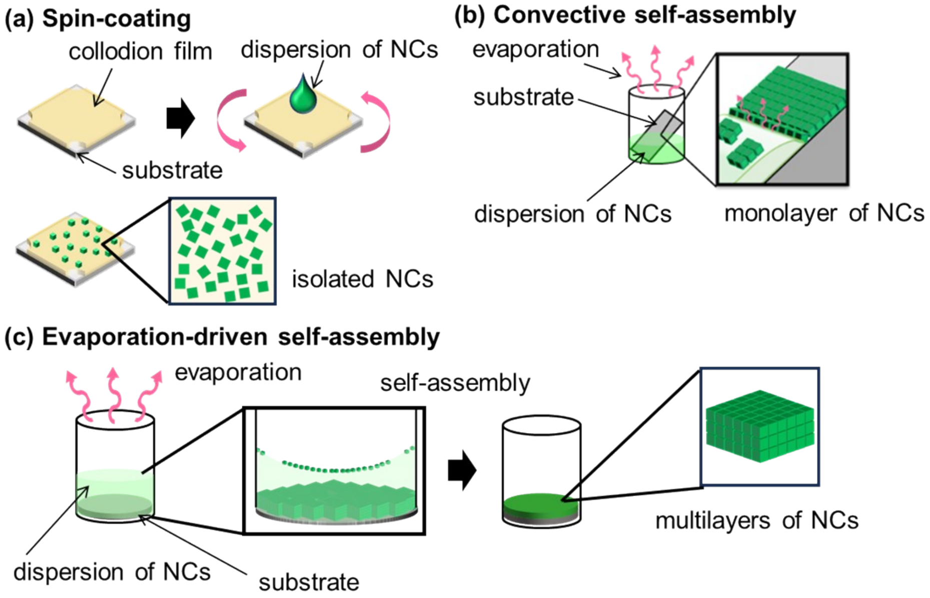

Figure S1. Schematic illustrations of preparation methods for various arrays of $CsPbBr_3$ NCs using organic medium.

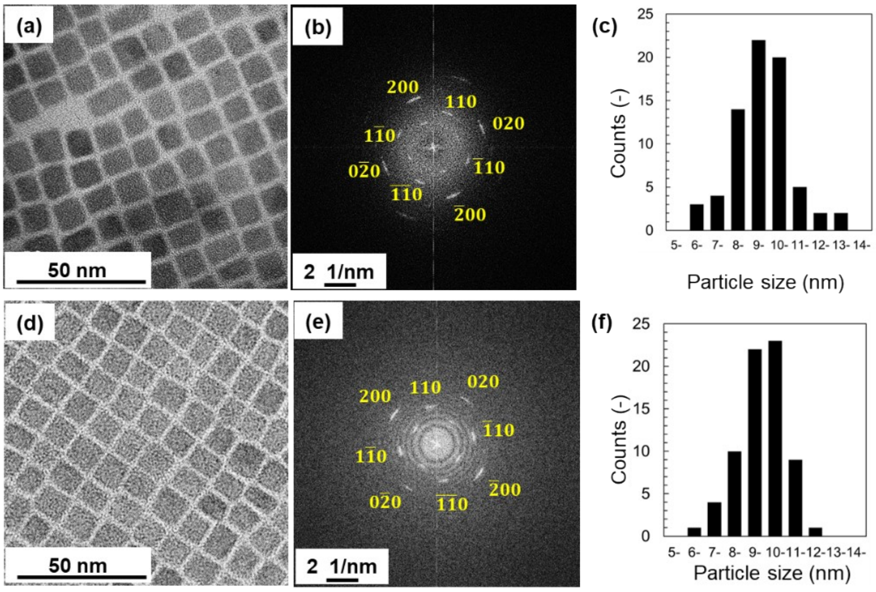

Figure S2. (a) TEM image, (b) FFT pattern, (c) size distribution of $CsPbBr_3$ NCs capped by OA / OLA, (d) TEM image, (e) FFT pattern, (f) size distribution of $CsPbBr_3$ NCs capped by DDAB.

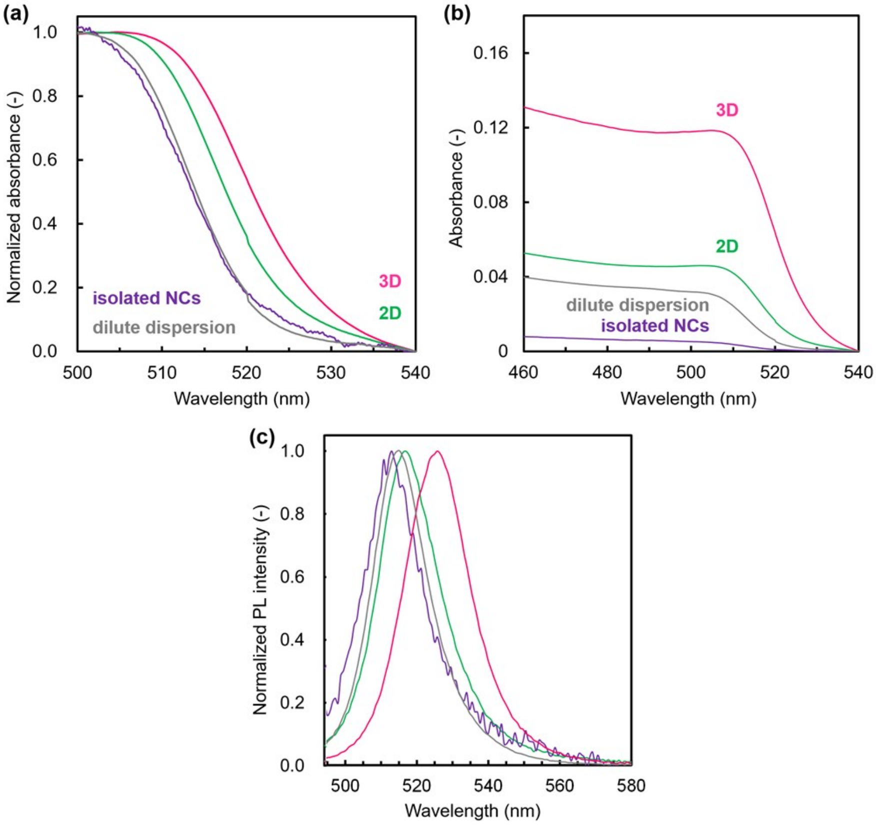

Figure S3. (a) Normalized and (b) absolute absorption spectra in UV-visible region and (c) PL spectra of $CsPbBr_3$ NCs in a dispersion, isolated NCs, and ordered arrays.

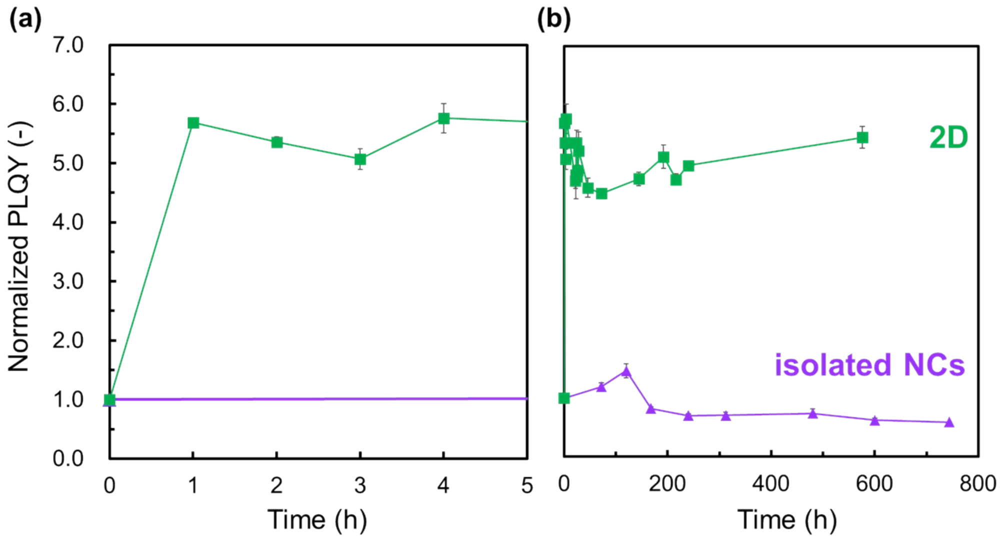

Figure S4 Time courses of PLQY of isolated NCs and 2D ordered assembled arrays under continuous photoirradiation at 468 nm within 800 h.

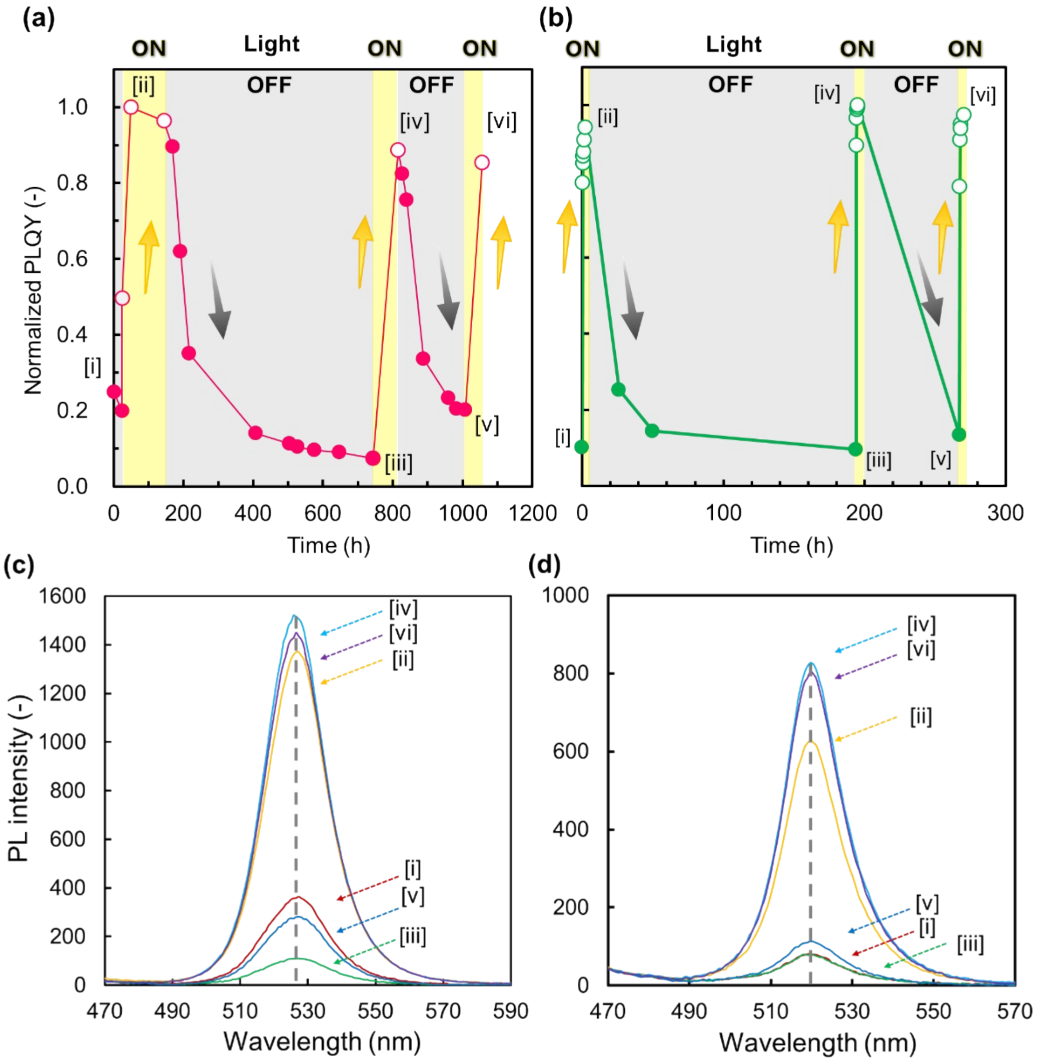

Figure S5. The PLQY change of the (a) 3D, (b) 2D ordered assembled arrays of $CsPbBr_3$ NCs with and without photoirradiation. PL spectra of (c) 3D, (d) 2D ordered assembled arrays of $CsPbBr_3$ NCs at the selected periods which correspond to the time shown in (a, b) the PLQY change.

(a, c) The PLQY of the 3D ordered assembled array was measured at the following period: [i] before photoirradiation, [ii] after 24 h with first photoirradiation, [iii] after 600 h with the first storing in the dark, [iv] after 72 h with second photoirradiation, [v] after 192 h with the second storing in the dark, [vi] after 48 h with third photoirradiation.

(b, d) The PLQY of the 2D ordered assembled array was measured at the following period: [i] before photoirradiation, [ii] after 2 h with first photoirradiation, [iii] after 144 h with the first storing in the dark, [iv] after 1.25 h with second photoirradiation, [v] after 72 h with the second storing in the dark, [vi] after 3 h with third photoirradiation.

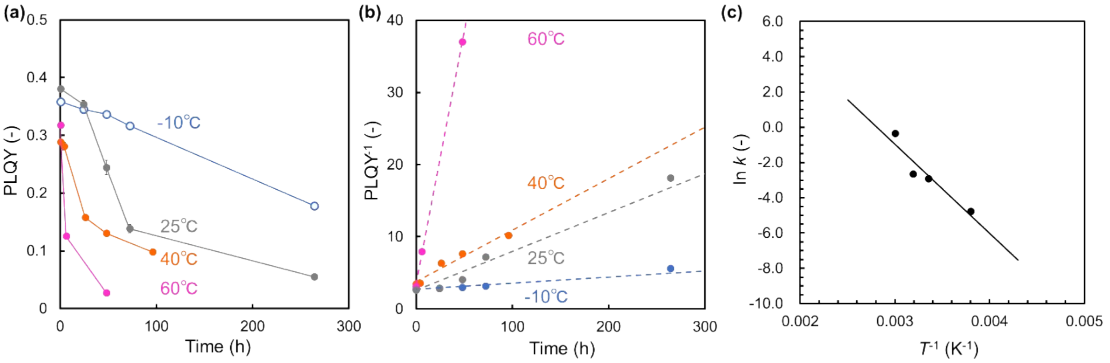

Figure S6. (a)The PLQY decrement in the dark at different temperature. 3D ordered assembled arrays of $CsPbBr_3$ NCs are stored in the dark at different temperature (T = –10°C, 25°C, 40°C, and 60°C) after increased to its maxima with photoirradiation.

Modeling the desorption kinetics using the pseudo-second-order equation[5,6].

$$\frac{dq_t}{dt} = k(q_e - q_t)^2$$

where k is the kinetic constant of pseudo-second order desorption, q_e and q_t are the amounts of adsorbate on the surface of NCs at equilibrium, and at time t, respectively.

Integrating the differential equation by applying the boundary conditions (q_t = 0 at t = 0, and q_t = q_t at t = t), the following equation is obtained:

$$\frac{t}{q_t} = \frac{1}{kq_e^2} + \frac{t}{q_e}$$

Based on this theoretical model, plotting $\frac{t}{q_t}$ against t shows linear relationship(b).

(c) Arrhenius plots and linear fitting in 3D ordered assembled arrays in the temperature range of 263-333 K using a pseudo-second-order Arrhenius equation.

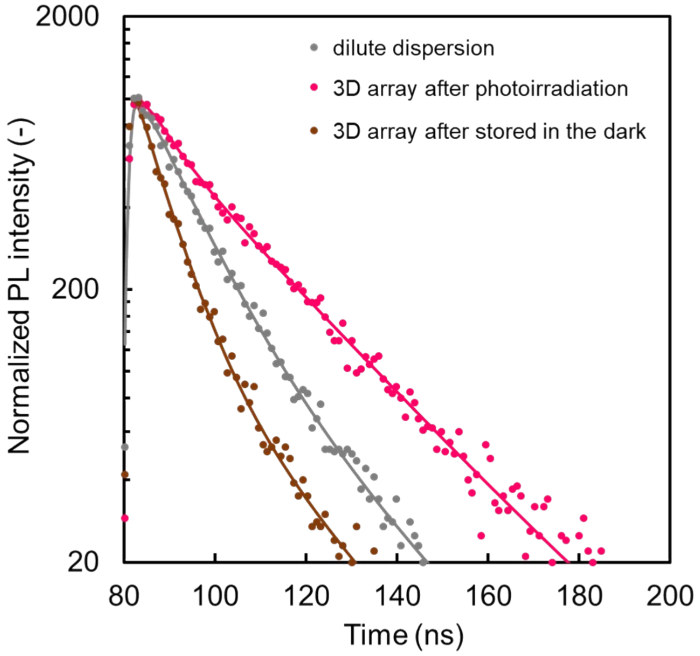

Figure S7. Time resolved PL decays of as-prepared dilute dispersion of $CsPbBr_3$ NC, 3D ordered arrays after photoirradiation, and after stored in the dark state. Solid lines indicate decay curves fitted by the following equation. (τ: PL lifetime, A: pre-exponential factor)

$I(t) = A_1 \exp(-\tau_1 t) + A_2 \exp(-\tau_2 t)$

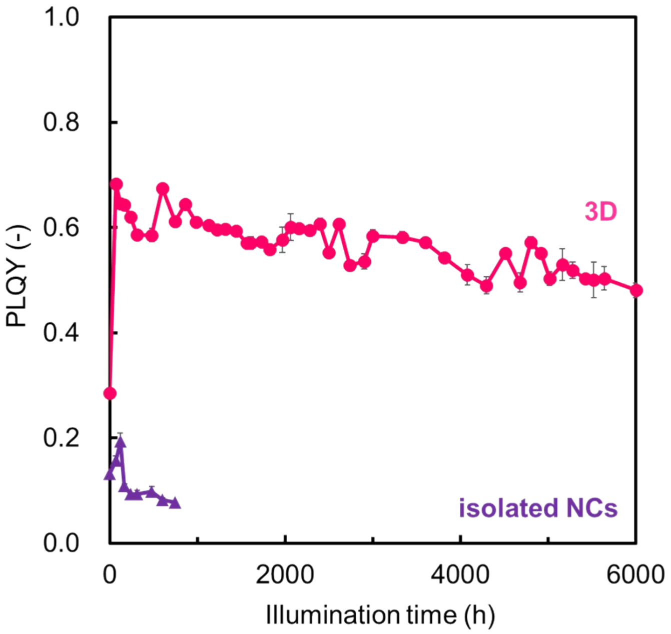

Figure S8. Time courses of PLQY of isolated NCs and 3D ordered assembled arrays under continuous blue-light illumination at 450 nm.

References

1 L. Protesescu, S. Yakunin, M. I. Bodnarchuk, F. Krieg, R. Caputo, C. H. Hendon, R. X. Yang, A. Walsh and M. V. Kovalenko, *Nano Lett.*, 2015, **15**, 3692–3696.

2 J. H. Park, A. Y. Lee, J. C. Yu, Y. S. Nam, Y. Choi, J. Park and M. H. Song, *ACS Appl. Mater. Interfaces*, 2019, **11**, 8428–8435.

3 M. Sasaki, S. Hashimoto, Y. Iso, Y. Oaki, T. Isobe and H. Imai, *Nanoscale Adv.*, 2023, **5**, 2553–2557.

4 K. Kidokoro, Y. Iso and T. Isobe, *J. Mater. Chem. C. Mater.*, 2019, **7**, 8546–8550.

5 M. Yalçin, A. Gürses, Ç. Doğar and M. SÖZBİLİr, *Adsorption*, 2005, **10**, 339–348.

6 B. Bai, Y. Wu and R. B. Grigg, *J. Phys. Chem. C*, 2009, **113**, 13772–13779.
